# 

**Published:** 1884-01

**Authors:** 


### INDEX.

PAGE.
Abnormal habits in animals ........................................ 241
A case of enteritis ........................................ 392
Act establishing a bureau of animal industry ........................................ 399
Actinomycosis discovered in American cattle ........................................ 111
Action of different proportions of chemical agents ........................................ 387
Acute gastro-enteritis in a bear ........................................ 113
Age marks, the ancients on equine ........................................ 19
American public health association ........................................ 111
    veterinary college ........................................ 294
        ninth annual commencement of ........................................ 188
Ancients on equine age marks ........................................ 19
Animals, abnormal habits in ........................................ 241
    castration ........................................ 294
    diseases, contagious, in Western New York ........................................ 330
    medicine as practised by ........................................ 113
    slaughtered, how inspected at the abattoir at Berlin ........................................ 88
    tuberculosis in wild ........................................ 116
Annual report of the Munich veterinary institute ........................................ 387
    royal commission ........................................ 86
    veterinary institute, Berlin ........................................ 85
        Vienna ........................................ 383
Art and science of veterinary medicine ........................................ 235

Bacillus of abortion ........................................ 381
Bacteria in the horse ........................................ 207
Bear, acute-gastro enteritis in a ........................................ 113
Bell, James, M.D., excisions of portions of the intestines in a dog ........................................ 272
Berlin, Prof., ophthalmoscopic appearance of the fundus of the eye of the
    horse ........................................ 32
    rupture of the sclerotica in a horse ........................................ 7, [illegible]8
Billings, Frank S., V.S., eclampsia parturientum ........................................ 117
    hæmatorrhacis or meningeal apoplexia ........................................ 195
    homœopathy ........................................ 309
    the Massachusetts veterinary association ........................................ 245
Board of health of Germany, the imperial ........................................ 263
Bone disease in monkeys ........................................ 115
Books and pamphlets received ........................................ 109, 205, 308, 397

94134

Bowler, G. W., M.D., enteritis in an elephant........ 149
Bronchitis fibrinosa in cattle, infectious........ 385
Bureau of animal industry........280, 376

Camel, diarrhœa in a........ 112
Cancer in domesticated animals........ 207
Case department........95, 195, 392
Cattle, actinomycosis discovered in American........ 111
fever, Texas........111, 213
rheumatism in........ 116
tuberculosis in........ 291
Cholesterin crystals in the vitreous humor of the eye in a horse........ 99
Clarke, William H., the ancients on equine age marks........ 19
Columbia veterinary college........ 92
suspension of........ 190
Comparative physiology of menstruation........58, 163, 254
Congenital misplacements in a dog........ 95
Conklin, W. A., D.V.S., some diseases of the ostrich........ 14
Contagious animal diseases in Western New York........ 330
Convention New York State........ 84
Correspondence........101, 193, 297, 391
Cow, a case of cramp in a........ 211
pneumocele in a........ 113
prolonged lactation in a........ 116
Crundall, Edward, V.S., contagious animal diseases in Western New York 330

Diarrhœa in a camel........ 112
Dick, Prof., statue........ 93
Diphtheria, propagation of by chickens........ 112
Diseases of the ostrich........ 14
sanitation and infectious........ 81
Does scarlet fever exist in the lower animals?........ 326
Dog, congenital misplacements in a........ 95
excision of portions of the intestines in a........ 272
hospital wanted........ 191
inoculation of with syphilis........ 206

Early history of veterinary medicine in the United States........ 25
Eclampsia parturientum........ 117
Editorial department........66, 172, 280, 376
notes........76, 190, 293, 383
Elephant, enteritis in an........ 149
Emesis in a horse........ 97
Empirics and the proposed veterinary association in Missouri........ 293
Enteritis, a case of........ 392
in an elephant........ 149
Equine scarlatinal virus, the use of........ 145
Excision of portions of the intestines in a dog........ 272

Experiments with reference to the development and regeneration of tendons . . . . 84
Eye in a horse, cholesterin crystals in the vitreous humor of the . . . . 99
of the horse, filaria in the vitreous humor of the . . . . 100
ophthalmoscopic appearance of the fundus of the . . . . 32

Fever, scarlet, in horses . . . . 1, 134
Filaria in the vitreous humor of the eye of the horse . . . . 100
Foot and mouth disease . . . . 178
a cowman attacked by . . . . 206
Frohner, Prof. Eugene, radical cure of umbilical hernia in a colt . . . . 392

Glanders, a contribution to the experimental diagnosis of . . . . 89
and farcy, treatment of by the dosimetric system of therapeutics. 209
in Newark . . . . 104
inoculation with the virus of . . . . 114
was it ? . . . . 197
Gratuitous treatment at clinics . . . . 83

Hæmatorrhacis or meningeal apoplexia . . . . 195
Hæmoglobinæmia toxica . . . . 361
Heroic treatment . . . . 116
History, early, of veterinary medicine in the United States . . . . 25
of tuberculosis . . . . 44, 338
Hogs, an examination of for trichinæ . . . . 90
cholera . . . . 111
Homœopathy . . . . 309
Horse, bacteria in a . . . . 207
cholesterin crystals in the vitreous humor of the eye of the . . . . 99
emesis in a . . . . 97
filaria in the vitreous humor of the eye of the . . . . 100
ophthalmoscopic appearance of the fundus of the eye of the . . . . 32
rupture of the sclerotica in a . . . . 98
scarlet fever in . . . . 1, 134

Infectious bronchitis fibrinosa in cattle . . . . 385
Inoculation of dogs with syphilis . . . . 206
syphilitic of monkeys . . . . 114
with virus of farcy glanders . . . . 114
International veterinary congress . . . . 73
Irido-choroiditis, a case of . . . . 90
Jennings, R., V.S., early history of veterinary medicine in the United States . . . . 25
Johne, Dr. Albert, history of tuberculosis, with special consideration of tuberculosis in cattle . . . . 44, 338

Lovell, R., V.S., was it glanders ? . . . . 197
Lowe, W. H., D.V.S., emesis in a horse . . . . 97

Massachusetts veterinary association ........ 245
Meat inspection at the Munich abattoir ........ 386
Meeting of the United States veterinary association ........ 185
Menstruation, comparative physiology of ........ 58, 163, 254
Michigan State veterinary medical association ........ 189
Miller, Joseph H., V.S., was the mare pregnant? ........ 96
Monkeys, bone disease in ........ 115
    syphilitic inoculation of ........ 114
Montreal veterinary college ........ 301
Munich veterinary institute ........ 387

Neuroretinitis optica ........ 153
News and miscellany ........ 116, 206
New York State convention ........ 84

Obituary ........ 206, 295
Oemler, Dr., the prevention of contagious pleuro-pneumonia in cattle ........ 351
Ohio State veterinary medical association ........ 397
Ophthalmoscopic appearance of the fundus of the eye of the horse ........ 32
Ostrich, some diseases of ........ 14
Oswald, F. L., M.D., abnormal habits in animals ........ 241

Pasteur's workshop, scenes in ........ 206
Peters, J. C., M.D., scarlet fever in horses ........ 1, 134
Plagiarism ........ 293
Pleuro-pneumonia ........ 381
    in cattle, the prevention of contagious ........ 351
Pneumocele in a cow ........ 113
Posology and therapeutics ........ 113
Prevention of contagious pleuro-pneumonia in cattle ........ 351
Progress of veterinary science ........ 111, 211
Protection of ignorance by the U. S. Government ........ 290

Radical cure of umbilical hernia in a colt ........ 372
Reports ........ 397
    annual of the royal commission ........ 86
    veterinary institute, Berlin ........ 85
Reviews ........ 107, 198, 305, 394
Rheumatism in cattle ........ 116
Royal commission, annual report of ........ 86
    veterinary schooi at Budapest ........ 182
Rupture of the sclerotica in a horse ........ 98

Salmon, D. E., D.V.M., protective virus investigations ........ 191
    Texas cattle fever ........ 213
Sanitation and infectious diseases ........ 81
Scarlatina equina ........ 78
Scarlatinal virus, the use of equine ........ 145
Scarlet fever, does it exist in the lower animals? ........ 326

Scarlet Fever in horses ........ 1, 134
the lower animals ........ 382
Scientific method iu veterinary education ........ 66
Sclerotica, rupture of the in a horse ........ 98
Seedy toe, the cause of ........ 211
Shoulder lameness ........ 116
Some recent investigations on germs ........ 402
Stickler, J. W., M.D., abstract of the investigations thus far made in the use of equine scarlatinal virus ........ 145

Tendons, experiments with reference to the development and regeneration of ........ 84
Texas cattle fever ........ 111, 213
To the profession ........ 376
Trichin-epidemics in Germany ........ 180
Trichinæ, an examination of hogs for ........ 90
in American pork ........ 283
Trichiniasis in Germany ........ 78
on the Jordan ........ 80
Tuberculosis, history of ........ 44, 338
in wild animals ........ 116
animals slaughtered at Augsburg ........ 292
cattle ........ 291
the contagion of ........ 112

Umbilical hernia in a colt ........ 392

Van Syckle, Alvah C., M. D., a case of Enteritis ........ 392
Veterinary association, Massachusetts ........ 245
semi-annual meeting of the United States ........ 185
college, American ........ 294
annual commencement ........ 188
Columbia ........ 92
suspension of ........ 190
Edinburgh, fire at ........ 190
Montreal ........ 301
congress, the international ........ 73
education, the scientific method in ........ 66
institute, Vienna, aunual report ........ 383
Bertine annual report ........ 85
medical association, Michigan ........ 189
New Jersey ........ 302
Ohio ........ 397
medicine, art, and science of ........ 235
department university, Penn ........ 212
in the United States ........ 172
early history of ........ 25
profession in the United States, to the ........ 76
sanitary science and preventive medicine ........ 150

Veterinary school, Milan ..... 383
Budapest ..... 182
science, progress of ..... 111, 211
Virus investigations, Dr. Salmon's protective ..... 191

Walley, Thomas, M.R.C.V.S., does scarlet fever exist in the lower animals? ..... 326
Ward, Robert, F.R.C.V.S., art and science of veterinary medicine ..... 235
veterinary sanitary science and preventive medicine ..... 150
Wiltshire, Alfred, M.D., comparative physiology of menstruation. 58, 163, 254

### BOOKS AND PAMPHLETS RECEIVED.

Annual Report of the Board of Regents of the Smithsonian Institution for the Year 1881. Washington, 1883.

Contagious Diseases of Domestic Animals—Investigations by Department of Agriculture. 8vo, pp. 271. Washington, 1883.

Johns Hopkins University Circulars.

Annals et Bulletin de la Sociétié de Medicine de Gand.

Journals.—La Clinica Veterinaria, Milano. The Veterinarian, London; The Veterinary Journal, London; Der Zoologische Garten, Frankfort;

Schweizerisches Archiv fur Thierheilkunde, und Thierzucht, Bern; Archiv fur Wissenschaftliche und Practische Thierheilkunde, Berlin; Journal de la Société contre L'Abus du Tabac, Paris; Tidskrift for Veterinar-Medicine, Stockholm; Giornale di Anatomia, Fisiologia e Patologia, degli Animali, Pisa; Il Medico Veterinario, Torino; El Ensayo Medico, Caracas; Wochenschrift fur Thierheilkunde und Viehzucht, Augsburg; Repertorium der Thierheilkunde, Stuttgart; The Quarterly Journal of Veterinary Science in India, Madras; Kansas City Review of Science and Industry; Chicago Medical Journal and Examiner; The Medical Record, New York; The College and Clinical Record, Philadelphia; Annals of Anatomy and Surgery, Brooklyn; New England Medical Monthly, Sandy Hook; Chicago Medical Review; Virginia Medical Monthly, Richmond; Nashville Journal of Medicine and Surgery; The Southern Clinic, Richmond; The Western Medical Reporter, Chicago; Journal of Cutaneous and Venereal Diseases, New York; Denver Medical Times; The St. Joseph Medical Herald; San Francisco Western Lancet; The Weekly Medical Review, St. Louis; The Blacksmith and Wheelwright, New York; National Live Stock Journal, Chicago; The Breeder's Gazette, Chicago; Cultivator and Country Gentleman, Albany; Indiana Farmer, Indianapolis; Truth, San Francisco; Weekly Drover's Journal, Chicago; The Chicago Tribune; The Medical Register, Philadelphia; Spirit of the Times, New York; United States Veterinary Journal, Chicago; Horseshoer and Hardware Journal, Chicago; The Polyclinic, Philadelphia; Medical World, Philadelphia; The New York Medical Journal.

